# Altered Heart Rate Variability During Rest in Schizophrenia: A State Marker

**DOI:** 10.7759/cureus.44145

**Published:** 2023-08-26

**Authors:** Anjum Datta, Sandeep Choudhary, Sunaina Soni, Rajesh Misra, Kiran Singh

**Affiliations:** 1 Physiology, Subharti Medical College and Associated Chhatrapati Shivaji Subharti Hospital, Meerut, IND; 2 Psychiatry, Subharti Medical College and Associated Chhatrapati Shivaji Subharti Hospital, Meerut, IND

**Keywords:** state markers of schizophrenia, sympathovagal balance, effect size, saps, sans, general linear model multivariate analysis, vagal tone, endophenotype, frequency domain, autonomic function testing

## Abstract

Background: Autonomic nervous system (ANS) imbalance has been reported in a number of psychiatric disorders such as depression, schizophrenia, panic disorder, etc. Autonomic dysfunction in schizophrenia has been associated with the symptoms and manifestation of psychosis. Heart rate variability (HRV) as a tool has been widely used to assess ANS activity and the effect of disease on the sympathovagal balance. Therefore, in the present study, HRV derived from electrocardiogram (ECG) lead II at rest was investigated in order to understand the changes in frequency domain measures in patients with schizophrenia and their first-degree relatives compared to healthy controls.

Methods: Twenty-five patients with schizophrenia, 24 first-degree relatives of patients, and 24 healthy controls (Diagnostic and Statistical Manual of Mental Disorders (DSM)-5; 18-45 years) were included in the study. HRV of the subjects was measured after five minutes of rest. ECG lead II was recorded for five minutes and HRV was analysed in the frequency domain: low frequency (LF), high frequency (HF), total power, and LF/HF ratio. HRV parameters and heart rate were statistically analysed for group comparisons using general linear model multivariate analysis.

Results: Patients had significantly higher minimum heart rate and lower HF (normalized units (nu)) compared to their first-degree relatives. A trend was observed in HF (nu) with the lowest in patients followed by healthy controls and first-degree relatives and LF/HF ratio was the highest in patients followed by healthy controls and first-degree relatives, although not statistically significant. No significant difference was found between first-degree relatives and healthy controls.

Conclusion: The alteration of HRV in schizophrenia could be attributed to reduction in vagal tone and sympathetic dominance, which in turn could serve as state markers of schizophrenia.

## Introduction

Autonomic dysfunction has long been associated with vulnerability to stress due to an impaired adaptation to environmental challenges [1]. Physiological measures of autonomic function may serve as indices to measure the extent of adaptation of an individual in order to regulate emotions and behaviour during changing environmental conditions [2].

The neurovisceral integration model suggests that heart rate variability (HRV) could be an index of central-peripheral neural feedback mechanisms during stress signifying cardiac vagal tone as a psychophysiological resource [3]. Autonomic nervous system (ANS) imbalance has been reported in a number of psychiatric disorders such as depression, schizophrenia, panic disorder, etc. [4,5].

Different authors have proposed that autonomic dysfunction in response to stress has a role in psychotic symptom formation, as schizophrenia could be characterized by a disruption of autonomic arousal and processing of stressful signals by amygdala-prefrontal circuits [6]. Kraepelin (1899) suggested autonomic alterations in patients with schizophrenia with increased sympathetic output, decreased parasympathetic activity, or both [7].

HRV has been widely used as a tool to assess ANS activity and the effect of disease on the sympathovagal balance in myocardial infarction, diabetic neuropathy, cardiac transplantation, myocardial dysfunction, tetraplegia, and renal failure. In recent years, there have been increased investigations of autonomic function for patients with schizophrenia as increased cardiovascular mortality has been reported owing to the relationship between symptoms of schizophrenia and cardiac autonomic irregularities [8,9].

Low HRV characterized by hyperactive sympathetic and/or hypoactive parasympathetic activity has been observed in schizophrenia. Its severity can be influenced by the psychotic state and duration of the disease [10]. Decreased vagal tone in schizophrenia is correlated with an increase in psychosis as assessed by the Positive and Negative Syndrome Scale (PANSS) [11]. HRV dysfunction may also be dependent on the phase of illness [12]. The metabolic effects of atypical antipsychotics pose a risk for weight gain and alteration of serum triglycerides and glycemic control [13]. It may give rise to a picture similar to metabolic syndrome, which results in reduced HRV. Both typical and atypical antipsychotics may increase cardiovascular risk and are associated with significant rates of lethal arrhythmias and instances of sudden death. Antipsychotic drugs affect the ANS via neuroleptic effects on various neurotransmitter receptors and autonomic imbalance may lead to increased morbidity and mortality in cardiovascular disease (CVD) [14,15].

Patients with schizophrenia also show a lack of activation in the medial prefrontal cortex, which might affect the inhibitory control over the autonomic function of the amygdala. This can lead to an exacerbation of arousal responses, which may result in low efferent vagal modulation and increased sympathetic activity [16]. Autonomic dysfunction in schizophrenia could present as decreased parasympathetic functioning with relatively normal sympathetic activation (~low frequency (LF)/high frequency (HF) ratio) [17] or elevated LF/HF ratio [18]. Two different conditions may exist: (a) increased sympathetic activation (high LF numerator) or (b) decreased parasympathetic activation (low HF denominator), which might result in sympathetic dominance.

Schizophrenia is a highly heritable disorder. Twin and adoption studies strongly suggest that genetic transmission accounts for most of the familial aggregation of schizophrenia [16]. An approach to the study of genetic transmission is the identification of intermediate phenotypes or endophenotypes in patients with schizophrenia and in their unaffected relatives [19]. First-degree relatives of patients with schizophrenia showed an attenuated and identical pattern in autonomic dysfunction as patients, with decreased vagal modulation of heart rate, decreased baroreflex sensitivity, and a similar pattern in regard to QT variability [16].

There are inconsistent and contradictory findings as to the underlying mechanisms of autonomic dysregulation in schizophrenia. Some studies attribute this to the psychopathology related to schizophrenia and some point towards the antipsychotic medication. Further, there is a need to establish biomarkers (state markers) based on HRV parameters in order to diagnose the autonomic changes even before clinical manifestations. The trait markers related to HRV in patients with schizophrenia and in healthy first-degree relatives may serve as an endophenotypic marker for schizophrenia, and hence a potential aid to discovering the genetic basis of this disorder.

Therefore, the present study aimed to investigate the HRV derived from ECG lead II at rest in order to understand the alterations in frequency domain indices (LF power represents sympathetic activity, HF power represents parasympathetic or vagal tone, and LF:HF ratio represents sympathovagal balance) in patients with schizophrenia and their first-degree relatives compared to healthy controls.

## Materials and methods

This was a cross-sectional, observational study conducted in the Department of Psychiatry at Subharti Medical College and Associated Chhatrapati Shivaji Subharti Hospital, Meerut, India. The study was approved by the Institutional Ethics Committee of Subharti Medical College and Associated Chhatrapati Shivaji Subharti Hospital, Swami Vivekanand Subharti University (approval number: SMC/EC/2016/22).

Subjects

Twenty-five patients with schizophrenia diagnosed as per the Diagnostic and Statistical Manual of Mental Disorders (DSM)-5, aged between 18-45 years, were included. Inclusion criteria were schizophrenia patients with an illness duration of less than five years, at least eight years of formal education, no hospitalization in the preceding two months, and on second-generation antipsychotics for 8-12 weeks. The sample size was estimated using the information available in the existing literature (means and standard deviation of different parameters) using nQuery Sample Size Software (GraphPad Software DBA Statistical Solutions, Boston, Massachusetts, United States) [20].Twenty-four first-degree relatives of patients and 24 age-, gender-, and education-matched healthy controls were also included and screened for psychopathology using the Mini-International Neuropsychiatric Interview, 6th Edition (MINI) [21]. The first-degree relatives of patients in the current study comprised siblings of the patients. Participants had no history of neurological and medical illness or substance use disorders.

Instruments

A semi-structured proforma was used for documenting the socio-demographic and clinical details. Various scales and questionnaires were administered including the Hindi version of the Mini-Mental State Examination (HMSE) to assess global cognition [22], the Scale for the Assessment of Negative Symptoms (SANS), and the Scale for the Assessment of Positive Symptoms (SAPS) for psychopathology assessment [23]. Handedness was assessed using the Edinburgh Handedness Inventory [24].

Procedure

HRV of subjects was measured at rest with a digitalized polygraph, RMS Polyrite D version 2.4 (Recorders & Medicare Systems Pvt. Ltd., Panchkula, Haryana, India) as per the standards laid by the Task Force of the European Society of Cardiology and the North American Society of Pacing and Electrophysiology in 1996 [25]. Participants were asked to lie down on a couch adjacent to the polygraph instrument, remain awake, and breathe normally. They were also asked to remove any metallic possessions that they might be wearing or carrying. ECG metallic electrodes were attached to the right arm, left arm and left leg. After five minutes of rest, ECG lead II was recorded for five minutes at a speed of 25 mm per second and voltage of 10 mm per mv to obtain short-term HRV. High and low filters were set at 99 and 0.1 Hz, respectively. Respiratory rate was obtained for all the participants [26]. The ECG data were used for offline analysis of HRV.

Data analysis

The ECG signals were analysed offline after visual checking for any artefacts or ectopic beats. HRV was analysed in the frequency domain (LF power, HF power, total power, and LF: HF ratio) using Kubios HRV software, version 2.2 (Kubios Oy, Kuopio, Finland). A recommendation of the Task Force was followed for analysis [25].

Statistical analysis

Statistical analysis was conducted using IBM SPSS Statistics for Windows, Version 20.0 (Released 2011; IBM Corp., Armonk, New York, United States). Data were checked for assumptions of normality using the Shapiro-Wilk test and homogeneity of variances using Levene’s test. Socio-demographic and clinical data were compared for groups using chi-square test adjusting p-value for multiple comparisons. HRV parameters and heart rate were analysed for group comparisons using general linear model multivariate analysis. The frequency domain parameters assessed for heart rate variability for comparisons between groups were LF (normalized units (nu)), HF (nu), LF/HF ratio, LF (ms^2^), HF (ms^2^), and total power along with maximum and minimum heart rate. Effect sizes were computed for significant findings between the groups by taking the difference in mean scores divided by the pooled standard deviation (Cohen’s d).

## Results

The demographic and clinical characteristics of patients with schizophrenia, their first-degree relatives, and healthy controls were homogeneous with respect to gender, age, years of education and ethnicity (Table 1). One-way ANOVA results for HMSE scores showed a significant difference between the groups (F (2, 70) = 20.857, p<0.001). Post hoc tests revealed that patients with schizophrenia had the lowest scores on HMSE followed by their first-degree relatives and healthy controls. Cohen’s d values for HMSE scores for patients vs. relatives (0.77), relatives vs. controls (1.37), and patients vs. controls (1.64) fall in the category of large effect size.

**Table 1:** Sociodemographic and clinical details of patients with schizophrenia, first-degree relatives, and healthy controls *p <0.01. S: patients with schizophrenia, R: first-degree relatives, C: healthy controls; SANS: Scale for the Assessment of Negative Symptoms; SAPS: Scale for the Assessment of Positive Symptoms; HMSE: Mini-Mental State Examination (Hindi version); INR: Indian Rupee

		Patients (N=25)	First-degree relatives (N=24)	Healthy controls (N=24)	p-value		
					S vs C	S vs R	R vs C
Age (years), mean (SD)		27.29 (5.98)	32.17 (8.09)	26.76 (6.08)	1	0.091	0.087
Gender, N (%)	Female	9 (36%)	8 (33.3%)	11 (45.83%)	0.482	0.956	0.474
	Male	16 (64%)	16 (66.6%)	13 (54.16%)			
Years of education, mean (SD)		13.27 (2.50)	13.48 (2.23)	14.68 (2.34)	0.062	0.931	0.160
Occupation, N (%)	Unemployed	7 (28%)	1 (4.16%)	0	0.007*	0.026	0.028
	Employed	9 (36%)	15 (62.5%)	9 (37.5%)	-	-	-
	Student	7 (28%)	5 (20.83%)	15 (62.5%)	-	-	-
	Housewife	2 (8%)	2 (8.33%)	0	-	-	-
	Retired	0	1 (4.16%)	0	-	-	-
Marital status, N (%)	Married	3 (12%)	11 (45.83%)	6 (25%)	0.163	0.009*	0.152
	Separated	3 (12%)	0	0	-	-	-
	Unmarried	19 (76%)	12 (50%)	18 (75%)	-	-	-
	Widower	0	1 (4.16%)	0	-	-	-
Monthly income (INR), N (%)	<5000	0	2 (8.33%)	0	0.004*	0.117	0.220
	5000-10000	7 (28%)	3 (12.5%)	6 (25%)	-	-	-
	10000-15000	12 (48%)	5 (20.83%)	1 (4.16%)	-	-	-
	15000-20000	6 (24%)	7 (29.16%)	3 (12.5%)	-	-	-
	>20000	0	7 (29.16%)	14 (58.33%)	-	-	-
Duration of illness (months), mean (SD)		45.35 (21.52)	-	-	-	-	-
Treatment duration (months), mean (SD)		38.27 (26.55)	-	-	-	-	-
SANS score, mean (SD)		66.51 (21.01)	-	-	-	-	-
SAPS score, mean (SD)		46.81 (19.84)	-	-	-	-	-
Medication, N	Second generation antipsychotic	25	-	-	-	-	-
	First generation antipsychotic	0					
Number of drugs, N	Monotherapy	25	-	-	-	-	-
	Poly-therapy	0					
HMSE score, mean (SD)		26.86 (2.89)	28.66 (1.56)	30.32 (0.69)	<0.001*	0.002*	0.012*

There was statistically no significant difference between the groups, F (18,124) = 1.224, p=0.252; Wilk’s Λ = 0.721, partial η2= 0.151. A significant difference was found for minimum heart rate, F (2,70) = 3.660, p=0.031, partial η2= 0.095 and HF (nu), F (2,70) = 3.439, p=0.038, partial η2= 0.089 with medium effect size.

**Figure 1:**
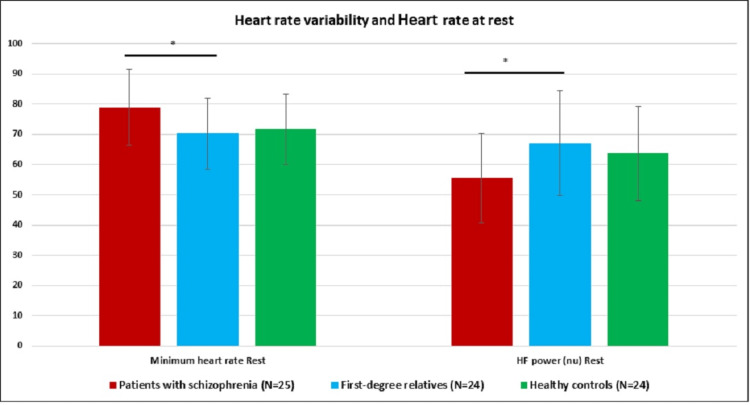
Heart rate variability and heart rate at rest compared between patients with schizophrenia, their first-degree relatives, and healthy controls Mean (SD) are represented; *p <0.05; Unit of heart rate: beats per minute HF: high frequency; nu: normalized units

Bonferroni post hoc test revealed that the patients had significantly higher minimum heart rate (p=0.041) and significantly lower HF (nu) compared to their first-degree relatives (p=0.040). A trend was observed in HF (nu) with the lowest in patients followed by healthy controls and first-degree relatives, and the LF/HF ratio was the highest in patients followed by healthy controls and first-degree relatives although not statistically significant. No significant difference was found between first-degree relatives and healthy controls (Table 2, Figure 1).

**Table 2:** Comparison of heart rate variability (HRV) parameters between groups at rest *p <0.05 S: patients with schizophrenia; R: first-degree relatives; C: healthy controls; nu: normalized units; LF: low frequency; HF: high frequency; LF/HF: low frequency/high frequency ratio.

	Patients with schizophrenia (N=25) Mean (SD)	First-degree relatives (N=24) Mean (SD)	Healthy controls (N=24) Mean (SD)	Post hoc test p-value		
				S vs C	S vs R	R vs C
Minimum heart rate (beats per minute)	78.96 (12.67)	70.25 (11.82)	71.79 (11.62)	0.124	0.041*	1
Maximum heart rate (beats per minute)	101.84 (16.48)	91.88 (18.67)	99.62 (40.13)	1	0.610	0.978
LF power (nu)	45.03 (14.47)	37.69 (19.63)	36.09 (15.68)	0.196	0.386	1
HF power (nu)	55.47 (14.66)	67.01 (17.32)	63.68 (15.59)	0.224	0.040*	1
LF/HF ratio	1.01 (0.92)	0.70 (0.69)	0.72 (0.73)	0.626	0.542	1
LF power (ms^2^)	283.92 (172.06)	282.55 (171.47)	225.93 (178.60)	0.743	1	0.791
HF power (ms^2^)	397.08 (271.94)	595.34 (395.84)	435.36 (370.66)	1	0.153	0.351
Total power (ms^2^)	716.50 (416.33)	908.86 (494.45)	686.02 (545.09)	1	0.515	0.353

## Discussion

In the present study, the HRV was investigated at rest in patients with schizophrenia and their first-degree relatives as compared to healthy controls in order to understand the pathophysiology of autonomic dysfunction in schizophrenia and to propose HRV-based state markers of schizophrenia. First-degree relatives of patients with schizophrenia were studied to identify HRV-based endophenotypic markers of schizophrenia in order to understand the genetic basis of the disorder.

Stress could act as a catalyst for the onset of schizophrenia when vulnerable individuals are exposed to it [27]. Existing literature suggests that in addition to stress reactivity to life stressors, hypothalamic-pituitary-adrenal (HPA) axis hyperactivity has also been reported among individuals with schizophrenia [28,29]. Autonomic dysfunction has been a core feature of the models proposed to explain vulnerability to stressors, due to an impaired adaptation to environmental challenges [1].

The main findings of the present study are that patients had significantly higher minimum heart rates and lower HF compared to their first-degree relatives. A trend was observed in HF with the lowest in patients followed by healthy controls and first-degree relatives and LF/HF ratio was highest in patients followed by healthy controls and first-degree relatives, although not statistically significant. No significant difference was found between first-degree relatives and healthy controls.

Increased heart rate has been reported long back by Emil Kraepelin in patients with schizophrenia which was suggestive of increased sympathetic output, decreased parasympathetic modulation, or both [7]. There have been contradictory and inconsistent findings on whether the increased heart rate is associated with the underlying pathophysiology of schizophrenia [30,31] or the effect of antipsychotic medication [32,33,34]. Previous literature has reported elevated heart rates in patients with schizophrenia compared to healthy controls [6,35,36]. However, in our study, heart rate was found to be elevated in patients with schizophrenia compared to their first-degree relatives with medium effect size, which is supported by the similar finding in previous studies suggesting increased heart rate in first-degree relatives of patients although less pronounced [16,37-40]. Increased resting heart rate could be related to impaired parasympathetic input to the heart in patients.

According to our study results, patients had significantly lower HF compared to their first-degree relatives. A trend was observed in HF with the lowest in patients followed by healthy controls and first-degree relatives and the LF/HF ratio was the highest in patients followed by healthy controls and first-degree relatives, although not statistically significant. Similar findings were reported by the previous studies in patients with schizophrenia compared to healthy controls [35,41-45]. On the contrary, Haigh et al. suggested greater LF power in controls compared to patients with schizophrenia and no group differences were observed in high power during an auditory EEG experiment [36], although not cognitively demanding. Further, medications were not found to have an effect on HRV parameters [36,46]. However, Clamor et al. reported that a higher dose of medication in patients with psychotic disorders (i.e., chlorpromazine equivalent) was moderately correlated with an increased heart rate and decreased HF HRV supporting potential medication add-on effects [35].

Higher heart rate and reduced LF power are believed to be associated with suppressed autonomic functioning, which in turn occurs in response to stress [47,48]. Therefore, studying markers of stress could be helpful for determining neurological and physiological health transdiagnostically throughout the lifespan and HRV provides us with a simple and cost-effective method of doing so. 

No significant difference was found between first-degree relatives and healthy controls. Therefore, we cannot comment on the endophenotypic marker based on our study findings. However, previous studies have reported findings that are contradictory to our results with attenuated and identical autonomic dysfunction, i.e. decreased parasympathetic activity in first-degree relatives of patients [16,37,38,49], which could serve as a physiological trait of individuals susceptible to developing schizophrenia.

Some limitations of the study include: patients recruited into our study had to be relatively stable and well enough to complete an extensive battery of clinical and ANS tests, which may potentially reduce the generalizability of our findings to a wider population including more severely ill and/or less cooperative patients. We did not use non-linear techniques to measure HRV. However, non-linear techniques may provide important information about alterations in the HR dynamics that are not detected by conventional spectral techniques in patients with schizophrenia. The patients excluded a subset of unmedicated or drug naïve patients with schizophrenia, and those who were recently hospitalized.

## Conclusions

The present study aimed to investigate the HRV at rest derived from ECG lead II in order to understand the alterations in frequency domain measures in patients with schizophrenia and their first-degree relatives as compared to healthy controls. According to our study results, disruption of sympathovagal balance was found in schizophrenia. Increased heart rate and lower HF power in patients at rest could be associated with reduction in vagal tone, which in turn could be attributed to sympathetic dominance. Therefore, the alterations in HRV at rest could serve as state markers of schizophrenia. 
