# Correction: Altered Heart Rate Variability During Rest in Schizophrenia: A State Marker

**DOI:** 10.7759/cureus.c176

**Published:** 2024-05-23

**Authors:** Anjum Datta, Sandeep Choudhary, Sunaina Soni, Rajesh Misra, Kiran Singh

**Affiliations:** 1 Physiology, Subharti Medical College and Associated Chhatrapati Shivaji Subharti Hospital, Meerut, IND; 2 Psychiatry, Subharti Medical College and Associated Chhatrapati Shivaji Subharti Hospital, Meerut, IND

This article has been corrected to accurately reflect the use of the Hindi version of the Mini-Mental State Examination. In the original version, the MMSE has been mentioned, but this has been changed to HMSE. The authors deeply regret that these errors were not identified and addressed prior to publication.

